# Factor XIII-A in Diseases: Role Beyond Blood Coagulation

**DOI:** 10.3390/ijms22031459

**Published:** 2021-02-01

**Authors:** Katalin Dull, Fruzsina Fazekas, Dániel Törőcsik

**Affiliations:** Department of Dermatology, Faculty of Medicine, University of Debrecen, Nagyerdei krt. 98, H4032 Debrecen, Hungary; kata.dull@med.unideb.hu (K.D.); fruzsi.fazekas28@gmail.com (F.F.)

**Keywords:** Factor XIII subunit A, chronic inflammatory diseases, malignancies

## Abstract

Multidisciplinary research from the last few decades has revealed that Factor XIII subunit A (FXIII-A) is not only involved in blood coagulation, but may have roles in various diseases. Here, we aim to summarize data from studies involving patients with mutations in the *F13A1* gene, performed in FXIII-A knock-out mice models, clinical and histological studies assessing correlations between diseases severity and FXIII-A levels, as well as from in vitro experiments. By providing a complex overview on its possible role in wound healing, chronic inflammatory bowel diseases, athe-rosclerosis, rheumatoid arthritis, chronic inflammatory lung diseases, chronic rhinosinusitis, solid tumors, hematological malignancies, and obesity, we also demonstrate how the field evolved from using FXIII-A as a marker to accept and understand its active role in inflammatory and malignant diseases.

## 1. Introduction

In the plasma, Factor XIII is present as a heterotetramer (FXIII-A_2_B_2_), consisting of two A subunits (FXIII-A) with catalytic activity and two B subunits (FXIII-B) that inhibit FXIII-A by binding to it. During the coagulation cascade, thrombin cleaves off the activation peptide from A subunit, the B subunit dissociates in a Ca^2+^-dependent manner, and FXIII-A becomes an active transglutaminase cross-linking fibrin strands, accounting for its role in stabilizing fibrin clots in the final stage of blood coagulation (reviewed in [1,2,3,4]).

Immunohistochemical detection later revealed that FXIII-A is also found intracellularly (cFXIII-A) in various cell types, where it may be activated without proteolytic cleavage by increased intracellular Ca^2+^ concentrations [5,6]. In the early embryonic life, mesenchymal histiocytes and hepatocytes [7] stained positive for FXIII-A; in adult life, megakaryocytes [8], platelets [9], monocytes [10,11], macrophages [10,12], dendritic cells [13], chondrocytes [14,15,16], osteoblasts [17], preadipocytes [18], corneal keratocytes [19], keratinocytes [20], fibroblasts [21], and sebocytes [22] all did as well. Importantly, detection of FXIII-A is still routinely used as a marker in the histological diagnosis of a wide range of inflammatory and malignant skin diseases (reviewed in [23]). Although some of the reported positive stainings were later found to be dependent on the applied antibody [22], while with the improvement of functional assays to distinguish macrophages from dendritic cells, FXIII-A became accepted as a marker of alternatively activated macrophages (a subset which is important in tissue remodeling and not in the elimination of bacteria [24]), rather than of dendritic cells [25,26,27]. These findings raised intriguing question on the possible role for FXIII-A both intracellularly and in (patho)physiological conditions beyond blood coagulation.

Factor XIII-A deficiency is mainly caused by mutations in the FXIII-A gene (95% of cases) [28]; while homozygous mutations usually result in symptoms related to an impaired blood coagulation, patients with heterozygous mutations are often asymptomatic. Identification of patients with decreased or even diminished activity of FXIII-A and patients with genetic variations (SNPs) in the gene *F13A1* [29], as well as the generation of genetically modified mouse models [30,31], has led the field into the era of proof-of-concept studies. Their characterization has demonstrated that indeed FXIII-A is not only involved in blood coagulation, but may have a role in wound healing and in basic immunological functions as well.

Implicating state-of-the art, unbiased research strategies and molecular methods into FXIII-A research brought another breakthrough and opened new vistas to define its relationship to diseases, many of which were not raised previously even at the level of speculation. Whole gene expression profiling has revealed that in alternatively activated macrophages, cFXIII-A may alter the expression of genes related to immune functions and to wound response [32]; it has also shown that FXIII-A shows abundant expression levels in adipose tissue, with a suggested role in adipose tissue development [33]. Protein detection confirmed its possible intracellular substrate in the pathogenesis of atherosclerosis [34]. Modern “gene hunting”, using whole exome sequencing, has identified mutations in FXIII-A to be the cause of familial dermatofibromas [35]. In addition, clinical studies have found and explained correlations with its altered expression levels and disease pathogenesis/severity in inflammatory diseases [36,37,38,39,40,41,42].

**Figure 1:**
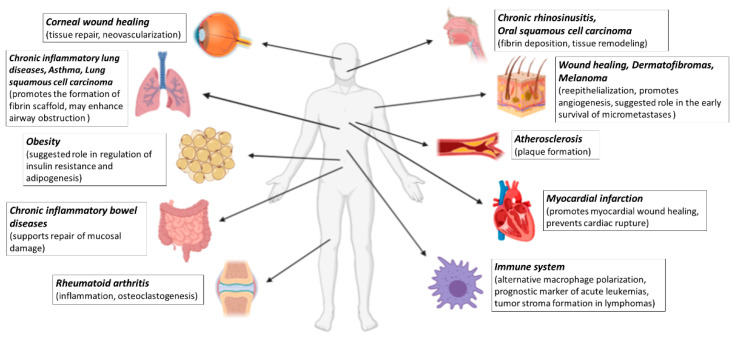
Summary overview of diseases discussed in this review in which FXIII-A may play a role. Created with BioRender.com.

In this review, we aim to summarize the available results and our recent understanding on the role of FXIII-A in various diseases (Figure 1).

## 2. FXIII-A in Diseases

### 2.1. FXIII-A in Wound Healing

Patients diagnosed with FXIII-A deficiency often show impaired tissue repair [43,44]. This phenomenon was characterized by Inbal et al. in a transgenic mice model with a targeted deletion of exon 7 in the *F13A1* gene, where delayed re-epithelialization and necrotized fissures were detected [45]. Importantly, when deficient mice were treated with recombinant FXIII-A, wound healing was normal, suggesting a pivotal role for FXIII-A from the plasma/extracellular space. The workgroup hypothesized that FXIII-A may have a regulatory role in multiple aspects of wound healing: stabilize fibrin clots and extracellular matrix, modulate monocyte functions, promote angiogenesis, and upregulate proangiogenic early growth response transcription factor 1 (Egr-1).

Angiogenesis and neovascularization also have a central role in corneal wound healing. Regarding FXIII-A in this process, Orosz et al., using 16 corneas from 9 donors, showed that corneal keratinocytes expressed FXIII-A, and measured elevated FXIII-A levels in human tears following full-thickness corneal transplantation. Their findings have suggested that FXIII-A in normal tears could be involved in the repair of micro-injuries, and the increased FXIII-A concentrations observed after corneal transplantation may promote the healing of surgical wounds. However, they also found that in higher concentrations, the effect of FXIII-A might become harmful by impairing fibrin elimination and enhancing neovascularization [46].

Nahrendorf et al. aimed to determine the relationship between FXIII-A activity and healing after myocardial infarction (MI) [47]. In a model of MI caused by left coronary artery ligation, they observed impaired wound healing and fatal rupture of the left ventricle in FXIII-deficient mice, while in wild-type mice a strong presence of FXIII-A positive cells was detected, with increased transglutaminase activity within the healing injured tissue. Replenishment of FXIII-A during the acute and subacute period restored survival rates, confirming that FXIII-A may have a role in the prevention of cardiac rupture. Further characterization of FXIII-A-deficient mice revealed an impaired inflammatory response, marked by lipopolysaccharide-induced CXC (cysteine-X-cysteine motif) chemokine (LIX)- and intercellular adhesion molecule-1 (ICAM)-independent neutrophil invasion, as well as enhanced degradation of the extracellular matrix with impaired collagen synthesis. However, FXIII-A re-substitution did not augment collagen synthesis in FXIII-A-deficient mice, suggesting a complex role for FXIII-A, in which its intracellular form might be involved as well.

### 2.2. FXIII-A in Chronic Inflammatory Bowel Diseases

Chronic inflammatory bowel diseases (CIBDs), e.g., Crohn’s disease (CD) and ulcerative colitis (UC), are characterized by large wound areas and ulcerations, with spontaneous hemorrhage in the intestines and the colon [48].

In an early study, Galloway et al. reported a markedly decreased Factor XIII-A serum level in 6 out of 13 patients with CIBD [49], which was later confirmed by several groups [50,51,52,53]. Studies further showed that FXIII-A levels were significantly lower in patients with active than in those with inactive CD, which suggests that FXIII-A is associated with disease activity [36,37]. However, in a study by Cougard et al., based on 129 patients’ data, although patients with CD had significantly lower FXIII-A serum levels when compared to controls, when CD patients with flare-ups were compared to ones in the remission phase, the difference did not reach the level of significance [54]. Moreover, there was no correlation between lower FXIII-A levels and the length of flare-ups.

Several polymorphisms are known in the *F13A1* gene [55], of which rs5985 is the most common one, changing valine with leucine at position 34 (Val34Leu) near the thrombin activation site, causing faster FXIII-A activation and rapid fibrin clot formation [56,57]. Indeed, the Val34Leu SNP of FXIII-A is associated with a reduced risk of venous thrombosis for homozygous carriers [58]. Saibeni et al. examined the Val34Leu polymorphism distribution and prevalence between 152 inflammatory bowel disease (IBD) patients and 130 healthy controls, and found no difference between IBD and healthy volunteers [59]. This indicates that the prothrombotic state described in IBD patients does not depend on the Val34Leu polymorphism. Similarly, Heliö et al. observed no significant differences in the Val34Leu polymorphism allele frequencies between 573 IBD patients and 142 controls [60].

When Soendergaard et al. evaluated the consequences of intestinal inflammation on resident mucosal macrophages, focusing on the level and distribution of FXIII-A using 67 tissue samples from patients with active UC [61], they observed a decreased number of FXIII-A/CD68/CD163-positive macrophages, suggesting a reduced phenotype of alternative macrophage polarization and a consequent loss of cFXIII-A, in parallel with decreased plasmatic FXIII-A (pFXIII-A) levels. The loss of cFXIII-A may impact migration and phagocytosis, and hence limit pathogen eradication in UC.

Andersson et al., by examining colon samples from wild-type and FXIII-A deficient mice following the induction of colitis, delivered results that have suggested that the loss of FXIII-A may actively contribute to the pathogenesis of colitis, and could play an important role in the repair of mucosal damage [38]. In their experiments, wild-type mice after colitis-challenge had significantly decreased pFXIII-A levels, thus supporting previous observations that pFXIII-A levels might be a reliable marker for active inflammatory colitis. When examining the differences in the tissue damage of the colon, 25% of the colon samples from FXIII-A-deficient mice showed ulceration, while only 5% of wild-type samples remained ulcerated in the colitis injury “resolution phase”.

Interestingly, some studies have suggested an important role also for the resident intestinal flora in the pathogenesis of colitis in both humans and mice [62,63], assuming that FXIII-A may support bacterial clearance by crosslinking bacterial surface proteins to fibrin, thus limiting colitis severity [62,64,65].

### 2.3. FXIII-A in Atherosclerosis

Migration of monocytes into the arterial wall, and their transformation into lipid-rich foam cells, is a pivotal step in the pathogenesis of atherosclerosis. Abdalla et al. showed that angiotensin II-induced cFXIII-A activation facilitates the covalent cross-links between type 1 angiotensin receptor monomers, resulting in enhanced signaling and desensitization. This finding was associated with increased adhesion of monocytes to the endothelium in ApoE-deficient mice [33], which is one of the earliest detectable changes in atherosclerotic lesions (reviewed in [66]).

Microarray experiments also found upregulated expression of FXIII-A mRNA in peripheral blood cells from five coronary artery disease patients [67]. As a possible explanation, in human monocytes FXIII-A was shown to be involved in processes related to atherogenesis, such as receptor-mediated phagocytosis [68] or the remodeling of small arteries after a reduction in blood flow in mice [69].

According to the results of Naito et al., FXIII-A cross-linked fibrin-enhanced vascular smooth muscle cell migration in vitro. Since the migration of vascular smooth muscle cells to the intima is a key event during the pathogenesis of atherosclerosis, this finding further strengthens the role of FXIII-A in the development of atherosclerosis [70].

### 2.4. FXIII-A in Rheumatoid Arthritis

Rheumatoid arthritis (RA) is a chronic inflammatory, autoimmune disorder, primarily affecting the joints, causing synovial hyperplasia and tissue destruction. As a result, irreversible cartilage and bone destruction may occur. A link between an altered blood coagulation system and RA has been reported [71,72,73,74], suggesting a direct correlation between reduced levels of coagulation factors, increased levels of fibrin degradation products, and RA disease progression by local inflammation and altered osteoclast function.

Raghu et al. examined the possible role for FXIII-A in RA pathogenesis. Previously, they had reported that fibrinogen may contribute to collagen-induced arthritis (CIA) disease progression in mice by binding the leukocyte integrin receptor αMβ2, thus inducing local inflammation [75]. They revealed that in FXIII-A-deficient mice, bone and cartilage erosion was diminished when compared with FXIII-A wild-type littermate controls [76]. Examining the knee joints of FXIII-A-deficient mice, an altered pattern of fibrin deposition with decreased numbers of inflammatory cells and reduced levels of IL6 (interleukin 6) and IL1β proinflammatory cytokines were observed. Interestingly, lower osteoclast numbers and activity linked to deficient receptor activator of NFκB ligand (RANKL)-induced osteoclast formation was also found in FXIII-A deficient mice. Providing further evidence, the pharmacological inhibition of transglutaminase activity diminished the development of arthritis and osteoclastogenesis in wild-type mice, suggesting that targeting FXIII-A (and its transglutaminase activity) may provide an attractive therapeutic strategy to control inflammation, cartilage, and bone destruction in RA.

### 2.5. FXIII-A in Chronic Inflammatory Lung Diseases

Bronchiolar and alveolar fibrin deposition is known as a hallmark of acute and chronic inflammatory lung diseases [77]. Katona et al. determined cellular and plasmatic FXIII-A levels and their correlation with levels of D-dimer, a breakdown product of cross-linked fibrin, from bronchoalveolar lavage fluid (BALF) of 39 pediatric patients with bronchoalveolar inflammation [39]. They found that cFXIII-A, pFXIII-A, and D-dimer levels were significantly elevated in patient samples when compared to control ones; moreover D-dimer levels correlated also with neutrophil counts. Based on their findings that in BALF samples, FXIII-A was exclusively expressed in alveolar macrophages, they concluded that FXIII-A, which could be an important regulator of fibrin turnover in the extravascular compartment, might be released from activated alveolar macrophages.

Esnault et al. examined the possible association between FXIII-A and asthma, finding that FXIII-A expression correlated with increased airflow limitation [40]. Analyzing FXIII-A mRNA and protein levels in the BALF from seven patients before and after an allergen challenge, they found significantly upregulated FXIII-A levels in brochoalveolar cells after allergen exposure. As FXIII-A mRNA expression levels were found to be positively correlated with a type 2 immune response and mRNA levels of CD207 (marker for dendritic cells) and CD209 (marker for dendritic cells and alternatively activated macrophages), immune cells were suggested to be the main source of FXIII-A also in the BALF of asthma patients, with a possible role in enhancing airway obstruction.

### 2.6. FXIII-A in Chronic Rhinosinusitis

Chronic rhinosinusitis (CRS) is a clinically heterogeneous group, characterized by persistent inflammation of the upper airways. Based on clinical symptoms and histological findings, CRS is classified into two groups: CRS without and with nasal polyps (CRSsNP and CRSwNP, respectively). While sinonasal tissue samples of CRSsNP patients show a predominant infiltration of neutrophils with the presence of Th1 (T helper cell type 1) cytokines, CRSwNP samples show eosinophilic infiltration and a Th2-based cytokine profile [78], which is a strong inducer of FXIII-A expression in alternatively activated macrophages [25].

In CRSwNP samples, excessive fibrin deposition and low D-dimer levels were detected, suggesting reduced fibrinolysis, which may contribute to tissue remodeling and the progression of the disease. However, in spite of the prominent inflammation, tissue samples of nasal polyps showed low levels of fibrosis [79]. Further findings, detecting increased FXIII-A levels in nasal polyp tissues of CRSwNP patients concluded that FXIII-A derived from alternatively activated macrophages might be involved in the formation of fibrin deposits in nasal polyps. Overexpression of FXIII-A therefore may lead to the acceleration of the coagulation cascade, resulting in fibrin deposition, tissue remodeling, edema, or pseudocyst formation of the submucosa, thus promoting disease progression. The results of the workgroup put forward that targeting FXIII-A production of alternatively activated macrophages might have therapeutic potential for CRSwNP patients [80].

### 2.7. FXIII-A in the Tumor Microenvironment

#### 2.7.1. Solid Tumors

As reviewed by Pollard [81], tumor-associated macrophages (TAMs) populate the microenvironment in most solid tumors, with a possible contribution to clinical outcomes by affecting several processes during tumorigenesis, from tumor initialization to the formation of metastases. Importantly, in a great number of tumors, TAMs (as a type of alternatively activated macrophages) have been reported to be FXIII-A positive [82,83,84]. Based on the fact that FXIII-A stabilizes fibrin networks, it is reasonable to suppose that (TAM-derived) FXIII-A may have an important role also in the formation of tumor matrix fibrin deposits, which may facilitate tumor matrix generation and tumor progression [85].

Palumbo et al. reported that the metastatic potential of intradermally injected B16-BL6 melanoma cells after 12–13 days was diminished in FXIII-A-deficient mice. They found that FXIII-A was not required for the growth of tumors or stroma formation, but supported the early survival of micrometastases by stabilizing fibrin/platelet thrombi associated with the newly formed micrometastases, thus limiting natural killer cell (NK)-mediated clearance of tumor cells. Moreover, the genetic elimination of FXIII-A transglutaminase activity significantly reduced lung and hematogenous metastases [86].

Vairaktaris et al. performed genetic analysis of oral squamous cell carcinoma samples from 130 patients. They found that the Val34Leu SNP occurred frequently, which may cause a thinner fibrin network due to an altered FXIII-A activation in carcinoma samples. Their findings suggested that the Val34Leu polymorphism is associated with an increased risk for oral squamous cell carcinoma, but not with its progression and metastasis [87].

Porrello et al. [41] identified a subset of lung squamous cell carcinoma tumors, characterized by the dense infiltration of inflammatory monocytes (IMs)/TAM precursors that correlate with poor survival, based on the examination of 491 samples. They showed that chemokine ligand 2 (CCL2)-mediated IMs expressed high levels of FXIII-A, which could promote fibrin cross-linking to create a scaffold for carcinoma cell invasion and metastases. The presence of cross-linked fibrin in the tumor microenvironment is correlated with poor survival.

#### 2.7.2. Dermatofibromas

Dermatofibromas (DF) are common, benign fibro-histiocytic tumors characterized by the presence of fibroblasts, macrophages, and multinucleated giant cells [88]. Firstly, Cerio et al. demonstrated by immunohistochemical staining of 30 patient samples that numerous dermal cells of DF samples react with anti-FXIII-A antibodies [89], which (together with the hematopoietic stem cell marker CD34) gained importance in the differential diagnosis for dermatofibrosarcoma (DFSP), where tumor cells were negative for FXIII-A and positive for CD34 [90,91,92].

In seven individuals from two unrelated families, with multiple dermatofibromas inherited in an autosomal dominant pattern, Supsrisunjai et al. revealed a missense variant in the FXIII-A gene (c.2036A>T; rs201302247) by using whole exome sequencing [35]. This mutation was the only variant in all affected individuals with multiple dermatofibromas. Moreover, they found that this mutation in FXIII-A led to reduced crosslinking activity, promoted cell proliferation via mitogen-activated protein kinase (MAPK) and phosphatidylinositol 3 kinase (PI3K) activation, altered collagen production, and showed a higher association with a4b1 integrins on fibroblasts compared to control samples. These findings provided solid evidence that FXIII-A is not only a useful immunohistochemical marker for dermatofibromas, but is also directly involved in the molecular pathophysiology of multiple dermatofibromas.

#### 2.7.3. Hematological Malignancies

##### Acute Leukemias

FXIII-A expression was observed in circulating monocytes of 48 acute non-lymphoid leukemia (ANLL) patients by Invernizzi et al. [93]. A positive correlation was shown between the presence of FXIII-A and the expression markers of the monocytes, suggesting that FXIII-A may be considered also as a molecular marker of monocyte differentiation. They also confirmed the previous findings of Ádány et al. [94] that its synthesis starts in the early stage of monocytopoiesis, along with alpha naphthyl acetate esterase (ANAE), sometimes earlier than CD14 (marker of the of the myelomonocyte lineage). Since its expression is directly related to the cell type and maturation degree, its detection may be a useful tool in the characterization of acute leukemia (AL) [95], alongside traditional and immunological markers.

On the basis of FXIII-A expression in 49 pediatric B-cell progenitor acute lymphoblastic leukemia (BCP-ALL) patients, Gyurina et al. identified new subgroups and described first B-cell precursor blasts as FXIII-A-expressing cells [96]. The three subgroups, according to FXIII-A cytoplasmic expression patterns of lymphoblasts as revealed by flow cytometry (FXIII-A-negative; FXIII-A dim: a uniformly positive population, but with lower mean fluorescence intensity than the positive control cells; and FXIII-A bright: uniformly positive population with higher fluorescence intensity than the control), had distinct gene expression profiles as well. The study of Kárai et al., based on 55 pediatric patient samples, found that the FXIII-A negative subgroup had and unfavorable disease outcome, with worse event-free and overall survival compared to patients with FXIII-A-positive blasts [42], while an excellent correlation was seen between FXIII-A positivity and long-term survival. It is hypothesized that in the absence of FXIII-A, the migration of lymphoblasts from the osteoblastic niche (inner surface of the bone cavity) to the vascular niche might be limited [97]. Since immuno- or chemotherapeutic drug-mediated cell death is more pronounced in the vascular niche, FXIII-A negative lymphoblasts retained in the bone cavity may survive more. In conclusion, FXIII-A expression could be a useful diagnostic marker to identify cases that require further genetic examination and a prognostic factor in childhood BCL-ALL.

##### Hodgkin Lymphoma

In the lymph nodes of Hodgkin lymphoma patients, where fibrin deposits are a general observation [98], Ádány et al., examining 12 Hodgkin lymphoma samples, identified a unique FXIII-A-expressing cell type with macrophage origin [99]. These cells showed macrophage marker Leu M3 and ANAE positivity, but they were negative for HLA-DR, AcP, ALP, and ATPase, suggesting that this FXIII-A-positive cell type is different from any other macrophage cell type identified earlier in human lymph nodes. The authors concluded that FXIII-A-positive macrophages in Hodgkin lymphoma samples may have a function in malignant cell proliferation.

### 2.8. FXIII-A and Obesity

A genome-wide association study identified *F13A1* as the gene with the most abundant expression levels in the white adipose tissue of obese individuals. In addition to this, seven SNPs in the *F13A1* gene were linked to obesity, suggesting that FXIII-A may have an important regulatory role in adipose tissue biology [100].

Further studies examining weight-discordant monozygotic twins in association with FXIII-A expression have found that FXIII-A was the only transglutaminase with significantly elevated expression levels in adipose tissue of the heavier twin. They found that FXIII-A mRNA levels increased with weight gain and were associated with pro-inflammatory, cell stress, and tissue remodeling pathways, supporting its role in adipose tissue expansion and inflammation linked to obesity. Interestingly, they have also found a positive correlation between the expression of FXIII-A and phospholipase 2A (PLA2G16) and mitogen-activated protein kinase 1 (MAPK1) genes; both genes are shown to be involved in lipolysis. Based on these findings, Kaartinen et al. suggested that FXIII-A could be an important therapeutic target to control adipose tissue inflammation linked to human obesity [33,101].

Investigating the role of transglutaminase activity in adipogenesis, Myneni et al. demonstrated, using FXIII-A-deficient mice, that differentiating preadipocytes have abundant transglutaminase activity, which may be derived from FXIII-A [18]. Importantly, besides fibrin, fibronectin expressed in white adipose tissue—where it may have a regulatory role in cell proliferation, differentiation, and adipogenesis [102,103,104,105]—is an important extracellular substrate for FXIII-A [106]. Myneni et al. showed that the FXIII-A expression in mouse 3T3-L1 cell-derived preadipocytes negatively regulates adipogenesis by increasing plasma fibronectin matrix assembly, promotes preadipocyte proliferation, and increases the pro-proliferative effects of insulin, thus suggesting a new role for FXIII-A in energy metabolism.

In a follow-up study, high-fat diet induced insulin resistance and hyperinsulinemia in wild-type but not in FXIII-A deficient mice. Moreover, FXIII-A deficient mice, despite gaining the same bodyweight as wild-type control animals, showed metabolically healthy obesity with healthy adipose tissue expansion, as marked by an increase in the size of adipocytes, proliferation, reduced white adipose tissue inflammation, and lower plasma triglyceride levels. Based on these findings, FXIII-A may have a regulatory function in obesity, and thus could be an important molecular target to improve the metabolic profile and regulate insulin resistance [107].

## 3. Conclusions

Although the number of inflammatory and malignant diseases in which FXIII-A is suggested to be involved has increased, in most cases our knowledge on its possible role in disease development is still at the level of speculation. FXIII-A may play a role as a coagulation factor, altering clot stabilization and bleeding, just like an enzyme in the extracellular space that is able to cross-link proteins, such as fibronectin, vitronectin, osteopontin, thrombospondin, and certain adhesive glycoprotein components of the extracellular matrix [108,109,110,111]. Furthermore, especially in diseases where alternatively activated macrophages are potentially involved, FXIII-A may have a possible role as an intracellular/secreted enzyme as well. Considering the wide range of diseases, questions remain open on how these features of FXIII-A, alone or in combination, may contribute to disease pathogenesis and progression, and perhaps may link diseases of which many have nothing else in common but FXIII-A. In providing answers, and further start points, not only basic and clinical research but registry-based [29] epidemiological studies should gain focus as well.

Another intriguing question is how FXIII-A could be used for therapies. While its application in dermal histopathological diagnosis has stood the test of time, in clinical practice FXIII-A is used only for substitution therapies of FXIII-A deficient patients [112]. Although the high cost of recombinant FXIII-A is indeed a limiting factor, trials are needed to test how the results of animal studies and in vitro experiments could be translated into human settings. Importantly, further therapeutic approaches should also be assessed, such as altering the levels and activity of FXIII-A in the local microenvironment. While developing and selectively delivering specific inhibitors for FXIII-A on site may modulate its unwanted effects [113], identifying stimuli that could be applied to regulate the production and secretion of cFXIII-A into the local microenvironment may utilize its beneficial ones.

In summary, although our knowledge has improved significantly, it appears that there are still ample ways, such as exploring new diseases and applying novel methods, to go on in order to reveal the (patho)physiological effect of FXIII-A in full, and importantly, how that can be applied in future therapies.

## Data Availability

Not applicable.

## Abbreviations

AcP	Acide phosphatase
AL	Acute leukemia
ALP	Alkaline phosphatase
ANAE	Alpha naphthyl acetate esterase
ANLL	Acute non-lymphoid leukemia
BALF	Bronchoalveolar lavage fluid
BCP-ALL	B-cell progenitor acute lymphoblastic leukemia
CCL2 C-C	Motif chemokine ligand 2
cFXIII-A	intracellular Factor XIII subunit A
CD	Crohn’s disease
CIA	Collagen-induced arthritis
CIBD	Chronic inflammatory bowel diseases
CRS	Chronic rhinosinusitis
CRSsNP	Chronic rhinosinusitis without nasal polyp
CRSwNP	Chronic rhinosinusitis with nasal polyp
DF	Dermatofibroma
DFSP	Dermatofibrosarcoma
Egr-1	Early growth response transcription factor 1
F13A	Factor XIII subunit A coding gene
FXIII-A	Factor XIII subunit A
FXIII-B	Factor XIII subunit B
HLA-DR	Human leukocyte antigen - DR isotype
ICAM	Intercellular adhesion molecule-1
IM	Inflammatory macrophage
LIX	Lipopolysaccharide-induced CXC chemokine
MAPK1	Mitogen-activated protein kinase 1
NK	Natural killer cells
pFXIII-A	Factor XIII subunit A in the plasma
PI3K	Phosphatidylinositol 3 kinase
PLA2G16	Phospholipase 2A
RA	Rheumatoid arthritis
RANKL	Receptor activator of NFκB ligand
SNP	Single nucleotide polymorphism
TAM	Tumor associated macrophage
WBC	White blood cell
AcP	Acide phosphatase
AL	Acute leukemia
ALP	Alkaline phosphatase
ANAE	Alpha naphthyl acetate esterase
ANLL	Acute non-lymphoid leukemia
BALF	Bronchoalveolar lavage fluid
BCP-ALL	B-cell progenitor acute lymphoblastic leukemia
CCL2 C-C	Motif chemokine ligand 2
cFXIII-A	intracellular Factor XIII subunit A
CD	Crohn’s disease
CIA	Collagen-induced arthritis
CIBD	Chronic inflammatory bowel diseases
CRS	Chronic rhinosinusitis
CRSsNP	Chronic rhinosinusitis without nasal polyp
CRSwNP	Chronic rhinosinusitis with nasal polyp
DF	Dermatofibroma
DFSP	Dermatofibrosarcoma
Egr-1	Early growth response transcription factor 1
F13A	Factor XIII subunit A coding gene
FXIII-A	Factor XIII subunit A
FXIII-B	Factor XIII subunit B
HLA-DR	Human leukocyte antigen - DR isotype
ICAM	Intercellular adhesion molecule-1
IM	Inflammatory macrophage
LIX	Lipopolysaccharide-induced CXC chemokine
MAPK1	Mitogen-activated protein kinase 1
NK	Natural killer cells
pFXIII-A	Factor XIII subunit A in the plasma
PI3K	Phosphatidylinositol 3 kinase
PLA2G16	Phospholipase 2A
RA	Rheumatoid arthritis
RANKL	Receptor activator of NFκB ligand
SNP	Single nucleotide polymorphism
TAM	Tumor associated macrophage
WBC	White blood cell

## References

[1] Muszbek L., Yee V.C., Hevessy Z. (1999). Blood coagulation factor XIII: Structure and function. Thromb. Res..

[2] Lorand L. (2001). Factor XIII: Structure, activation, and interactions with fibrinogen and fibrin. Ann. N. Y. Acad. Sci..

[3] Lorand L. (2005). Factor XIII and the clotting of fibrinogen: From basic research to medicine. J. Thromb. Haemost..

[4] Ariëns R.A.S., Lai T.S., Weisel J.W., Greenberg C.S., Grant P.J. (2002). Role of factor XIII in fibrin clot formation and effects of genetic polymorphisms. Blood.

[5] Polgar J., Hidasi V., Muszbek L. (1990). Non-proteolytic activation of cellular protransglutaminase (placenta macrophage Factor XIII). Biochem. J..

[6] Muszbek L., Polgár J., Boda Z. (1993). Platelet factor XIII becomes active without the release of activation peptide during platelet activation. Thromb. Haemost..

[7] Kappelmayer J., Bacskó G., Birinyi L., Zákány R., Kelemen E., Adány R. (1995). Consecutive appearance of coagulation factor XIII subunit A in macrophages, megakaryocytes, and liver cells during early human development. Blood.

[8] Kiesselbach T.H., Wagner R.H. (1972). Demonstration of Factor XIII in human megakaryocytes by a fluorescent antibody technique. Ann. N. Y. Acad. Sci..

[9] Kiesselbach T.H., Wagner R.H. (1966). Fibrin-stabilizing factor: A thrombin-labile platelet protein. Am. J. Physiol..

[10] Henriksson P., Becker S., Lynch G., MacDonagh J. (1985). Identification of intracellular factor XIII in human monocytes and macrophages. J. Clin. Invest..

[11] Muszbek L., Adány R., Szegedi G., Polgár J., Kávai M. (1985). Factor XIII of blood coagulation in human monocytes. Thromb. Res..

[12] Adány R., Belkin A., Vasilevskaya T., Muszbek L. (1985). Identification of blood coagulation factor XIII in human peritoneal macrophages. Eur. J. Cell Biol..

[13] Nestle F.O., Zheng X.G., Thompson C.B., Turka L.A., Nickoloff B.J. (1993). Characterization of dermal dendritic cells obtained from normal human skin reveals phenotypic and functionally distinctive subsets. J. Immunol..

[14] Nurminskaya M., Linsenmayer T.F. (1996). Identification and characterization of up-regulated genes during chondrocyte hypertrophy. Dev. Dyn..

[15] Nurminskaya M., Magee C., Nurminsky D., Linsenmayer T.F. (1998). Plasma transglutaminase in hypertrophic chondrocytes: Expression and cell-specific intracellular activation produce cell death and externalization. J. Cell Biol..

[16] Rosenthal A.K., Masuda I., Gohr C.M., Derfus B.A., Le M. (2001). The transglutaminase, Factor XIIIA, is present in articular chondrocytes. Osteoarthr. Cartil..

[17] Nurminskaya M., Kaartinen M.T. (2006). Transglutaminases in mineralized tissues. Front. Biosci..

[18] Myneni V.D., Hitomi K., Kaartinen M.T. (2014). Factor XIII-A transglutaminase acts as a switch between preadipocyte proliferation and differentiation. Blood.

[19] Orosz Z.Z., Bárdos H., Shemirani A.H., Debreceni I.B., Lassila R., Riikonen A.S., Kremer Hovinga J.A., Seiler T.G., van Dorland H.A., Schroeder V. (2019). Cellular factor XIII, a transglutaminase in human corneal keratocytes. Int. J. Mol. Sci..

[20] Ando Y., Imamura S., Kannagi R. (1990). Human epidermis contains coagulation factor XIII. Arch. Dermatol. Res..

[21] Nemeth A.J., Penneys N.S. (1989). Factor XIIIa is expressed by fibroblasts in fibrovascular tumors. J. Cutan. Pathol..

[22] Uhlenhake E.E., Clark L.N., Smoller B.R., Shalin S.C., Gardner J.M. (2016). Nuclear factor XIIIa staining (clone AC-1A1 mouse monoclonal) is a sensitive and specific marker to discriminate sebaceous proliferations from other cutaneous clear cell neoplasms. J. Cutan. Pathol..

[23] Paragh L., Törocsik D. (2017). Factor XIII Subunit A in the Skin: Applications in Diagnosis and Treatment. Biomed Res. Int..

[24] Gordon S., Taylor P.R. (2005). Monocyte and macrophage heterogeneity. Nat. Rev. Immunol..

[25] Töröcsik D., Bárdos H., Nagy L., Adány R. (2005). Identification of factor XIII-A as a marker of alternative macrophage activation. Cell. Mol. Life Sci..

[26] Zaba L.C., Fuentes-Duculan J., Steinman R.M., Krueger J.G., Lowes M.A. (2007). Normal human dermis contains distinct populations of CD11c +BDCA-1+ dendritic cells and CD163+FXIIIA + macrophages. J. Clin. Invest..

[27] Töröcsik D., Bárdos H., Hatalyák Z., Dezs B., Losonczy G., Paragh L., Péter Z., Balázs M., Remenyik E., Ádány R. (2014). Detection of factor XIII-A is a valuable tool for distinguishing dendritic cells and tissue macrophages in granuloma annulare and necrobiosis lipoidica. J. Eur. Acad. Dermatology Venereol..

[28] Ivaskevicius V., Biswas A., Bevans C., Schroeder V., Kohler H.P., Rott H., Halimeh S., Petrides P.E., Lenk H., Krause M. (2010). Identification of eight novel coagulation factor XIII subunit A mutations: Implied consequences for structure and function. Haematologica.

[29] Ivaskevicius V., Seitz R., Kohler H.P., Schroeder V., Muszbek L., Ariens R.A.S., Seifried E., Oldenburg J. (2007). International registry on factor XIII deficiency: A basis formed mostly on European data. Thromb. Haemost..

[30] Lauer P., Metzner H.J., Zettlmeissl G., Li M., Smith A.G., Lathe R., Dickneite G. (2002). Targeted inactivation of the mouse locus encoding coagulation factor XIII-A: Hemostatic abnormalities in mutant mice and characterization of the coagulation deficit. Thromb. Haemost..

[31] Griffin K., Simpson K., Beckers C., Brown J., Vacher J., Ouwehand W., Alexander W., Pease R., Grant P. (2015). Use of a novel floxed mouse to characterise the cellular source of plasma coagulation FXIII-A. Lancet.

[32] Töröcsik D., Szeles L., Paragh G.J., Rakosy Z., Bardos H., Nagy L., Balazs M., Inbal A., Adány R. (2010). Factor XIII-A is involved in the regulation of gene expression in alternatively activated human macrophages. Thromb. Haemost..

[33] Kaartinen M.T., Arora M., Heinonen S., Rissanen A., Kaprio J., Pietiläinen K.H. (2020). Transglutaminases and Obesity in Humans: Association of F13A1 to Adipocyte Hypertrophy and Adipose Tissue Immune Response. Int. J. Mol. Sci..

[34] Abdalla S., Lother H., Langer A., El Faramawy Y., Quitterer U. (2004). Factor XIIIA transglutaminase crosslinks AT1 receptor dimers of monocytes at the onset of atherosclerosis. Cell.

[35] Supsrisunjai C., Hsu C.K., Michael M., Duval C., Lee J.Y.W., Yang H.S., Huang H.Y., Chaikul T., Onoufriadis A., Steiner R.A. (2020). Coagulation Factor XIII-A Subunit Missense Mutation in the Pathobiology of Autosomal Dominant Multiple Dermatofibromas. J. Invest. Dermatol..

[36] Wisén O., Gårdlund B. (1988). Hemostasis in crohn’s disease: Low factor XIII levels in active disease. Scand. J. Gastroenterol..

[37] Hudson M., Wakefield A.J., Hutton R.A., Sankey E.A., Dhillon A.P., More L., Sim R., Pounder R.E. (1993). Factor XIIIA subunit and Crohn’s disease. Gut.

[38] Andersson C., Kvist P.H., McElhinney K., Baylis R., Gram L.K., Pelzer H., Lauritzen B., Holm T.L., Hogan S., Wu D. (2015). Factor XIII transglutaminase supports the resolution of mucosal damage in experimental colitis. PLoS ONE.

[39] Katona É., Nagy B., Kappelmayer J., Baktai G., Kovács L., Márialigeti T., Dezso B., Muszbek L. (2005). Factor XIII in bronchoalveolar lavage fluid from children with chronic bronchoalveolar inflammation. J. Thromb. Haemost..

[40] Esnault S., Kelly E.A., Sorkness R.L., Evans M.D., Busse W.W., Jarjour N.N. (2016). Airway factor XIII associates with type 2 inflammation and airway obstruction in asthmatic patients. J. Allergy Clin. Immunol..

[41] Porrello A., Leslie P.L., Harrison E.B., Gorentla B.K., Kattula S., Ghosh S.K., Azam S.H., Holtzhausen A., Chao Y.L., Hayward M.C. (2018). Factor XIIIA-expressing inflammatory monocytes promote lung squamous cancer through fibrin cross-linking. Nat. Commun..

[42] Kárai B., Hevessy Z., Szánthó E., Csáthy L., Ujfalusi A., Gyurina K., Szegedi I., Kappelmayer J., Kiss C. (2018). Expression of Coagulation Factor XIII Subunit A Correlates with Outcome in Childhood Acute Lymphoblastic Leukemia. Pathol. Oncol. Res..

[43] Miloszewski K.J.A., Anwar R. (1999). Factor XIII deficiency. Br. J. Haematol..

[44] Board P.G., Lososky M.S., Miloszewski K.J.A. (1993). Factor XIII: Inherited and acquired deficiency. Blood Rev..

[45] Inbal A., Lubetsky A., Krapp T., Castel D., Shaish A., Dickneitte G., Modis L., Muszbek L., Inbal A. (2005). Impaired wound healing in factor XIII deficient mice. Thromb. Haemost..

[46] Orosz Z.Z., Katona É., Facskó A., Módis L., Muszbek L., Berta A. (2011). Factor XIII subunits in human tears; their highly elevated levels following penetrating keratoplasty. Clin. Chim. Acta.

[47] Nahrendorf M., Hu K., Frantz S., Jaffer F.A., Tung C.H., Hiller K.H., Voll S., Nordbeck P., Sosnovik D., Gattenlöhner S. (2006). Factor XIII deficiency causes cardiac rupture, impairs wound healing, and aggravates cardiac remodeling in mice with myocardial infarction. Circulation.

[48] Lorenz R., Olbert P., Born P. (1996). Factor XIII in Chronic Inflammatory Bowel Diseases. Semin. Thromb. Hemost..

[49] Galloway M.J., Mackie M.J., McVerry B.A. (1983). Reduced levels of factor XI11 in patients with chronic inflammatory bowel disease. Clin. Lab. Haematol..

[50] Suzuki R., Toda H., Takamura Y. (1989). Dynamics of blood coagulation factor XIII in ulcerative colitis and preliminary study of the factor XIII concentrate. Blut.

[51] D’Argenio G., Biancone L., Cosenza V., Della Valle N., D’Armiento F.P., Boirivant M., Pallone F., Mazzacca G. (1995). Transglutaminases in Crohn’s disease. Gut.

[52] Chamouard P., Grunebaum L., Wiesel M.L., Sibilia J., Coumaros G., Wittersheim C., Baumann R., Cazenave J.P. (1998). Significance of diminished factor XIII in Crohn’s disease. Am. J. Gastroenterol..

[53] Seitz R., Leugner F., Katschinski M., Immel A., Kraus M., Egbring R., Göke B. (1994). Ulcerative colitis and crohn’s disease: Factor XIII, inflammation and haemostasis. Digestion.

[54] Cougard P.A., Desjeux A., Vitton V., Baumstarck-Barrau K., Lesavre N., Grimaud J.C. (2012). The usefulness of factor XIII levels in Crohn’s disease. J. Crohn’s Colitis.

[55] Bagoly Z., Koncz Z., Hársfalvi J., Muszbek L. (2012). Factor XIII, clot structure, thrombosis. Thromb. Res..

[56] Schroeder V., Chatterjee T., Kohler H.P. (2001). Influence of blood coagulation factor XIII and FXIII Val34Leu on plasma clot formation measured by thrombelastography. Thromb. Res..

[57] Ariëns R.A., Philippou H., Nagaswami C., Weisel J.W., Lane D.A., Grant P.J. (2000). The factor XIII V34L polymorphism accelerates thrombin activation of factor XIII and affects cross-linked fibrin structure. Blood.

[58] Bernstein C.N., Sargent M., Vos H.L., Rosendaal F.R. (2007). Mutations in clotting factors and inflammatory bowel disease. Am. J. Gastroenterol..

[59] Saibeni S., Vecchi M., Faioni E.M., Franchi F., Rondonotti E., Borsi G., de Franchis R. (2003). Val34Leu factor XIII polymorphism in Italian patients with inflammatory bowel disease. Dig. Liver Dis..

[60] Heliö T., Wartiovaara U., Halme L., Turunen U.M., Mikkola H., Palotie A., Färkkilä M., Kontula K. (1999). Arg506Gln factor V mutation and Va134Leu factor XIII polymorphism in Finnish patients with inflammatory bowel disease. Scand. J. Gastroenterol..

[61] Soendergaard C., Kvist P.H., Seidelin J.B., Pelzer H., Nielsen O.H. (2016). Systemic and intestinal levels of factor XIII-A: The impact of inflammation on expression in macrophage subtypes. J. Gastroenterol..

[62] Saleh M., Trinchieri G. (2011). Innate immune mechanisms of colitis and colitis-associated colorectal cancer. Nat. Rev. Immunol..

[63] Nagalingam N.A., Lynch S.V. (2012). Role of the microbiota in inflammatory bowel diseases. Inflamm. Bowel Dis..

[64] Degen J.L., Bugge T.H., Goguen J.D. (2007). Fibrin and fibrinolysis in infection and host defense. J. Thromb. Haemost..

[65] Loof T.G., Mörgelin M., Johansson L., Oehmcke S., Olin A.I., Dickneite G., Norrby-Teglund A., Theopold U., Herwald H. (2011). Coagulation, an ancestral serine protease cascade, exerts a novel function in early immune defense. Blood.

[66] Galkina E., Ley K. (2009). Immune and Inflammatory Mechanisms of Atherosclerosis. Annu. Rev. Immunol..

[67] Ma J., Liew C.-C. (2003). Gene profiling identifies secreted protein transcripts from peripheral blood cells in coronary artery disease. J. Mol. Cell. Cardiol..

[68] Sárváry A., Szucs S., Balogh I., Becsky Á., Bárdos H., Kávai M., Seligsohn U., Egbring R., Lopaciuk S., Muszbek L. (2004). Possible role of factor XIII subunit A in Fcγ and complement receptor-mediated phagocytosis. Cell. Immunol..

[69] Bakker E.N.T.P., Pistea A., Spaan J.A.E., Rolf T., De Vries C.J., Van Rooijen N., Candi E., Vanbavel E. (2006). Flow-dependent remodeling of small arteries in mice deficient for tissue-type transglutaminase: Possible compensation by macrophage-derived factor XIII. Circ. Res..

[70] Naito M., Nomura H., Iguchi A., Thompson W.D., Smith E.B. (1998). Effect of crosslinking by factor XIIIa on the migration of vascular smooth muscle cells into fibrin gels. Thromb. Res..

[71] Zacharski L.R., Brown F.E., Memoli V.A., Kisiel W., Kudryk B.J., Rousseau S.M., Hunt J.A., Dunwiddie C., Nutt E.M. (1992). Pathways of coagulation activation in situ in rheumatoid synovial tissue. Clin. Immunol. Immunopathol..

[72] Busso N., Hamilton J.A. (2002). Extravascular Coagulation and the Plasminogen Activator/Plasmin System in Rheumatoid Arthritis. Arthritis Rheum..

[73] Hoppe B., Dörner T. (2012). Coagulation and the fibrin network in rheumatic disease: A role beyond haemostasis. Nat. Rev. Rheumatol..

[74] Mousa A., Cui C., Song A., Myneni V.D., Sun H., Li J.J., Murshed M., Melino G., Kaartinen M.T. (2017). Transglutaminases factor XIII-A and TG2 regulate resorption, adipogenesis and plasma fibronectin homeostasis in bone and bone marrow. Cell Death Differ..

[75] Raghu H., Flick M.J. (2011). Targeting the Coagulation Factor Fibrinogen for Arthritis Therapy. Curr. Pharm. Biotechnol..

[76] Raghu H., Cruz C., Rewerts C.L., Frederick M.D., Thornton S., Mullins E.S., Schoenecker J.G., Degen J.L., Flick M.J. (2015). Transglutaminase factor XIII promotes arthritis through mechanisms linked to inflammation and bone erosion. Blood.

[77] Idell S., Mazar A.P., Bitterman P., Mohla S., Harabin A.L. (2001). Fibrin turnover in lung inflammation and neoplasia. Am. J. Respir. Crit. Care Med..

[78] Van Zele T., Claeys S., Gevaert P., Van Maele G., Holtappels G., Van Cauwenberge P., Bachert C. (2006). Differentiation of chronic sinus diseases by measurement of inflammatory mediators. Allergy Eur. J. Allergy Clin. Immunol..

[79] Takabayashi T., Kato A., Peters A.T., Hulse K.E., Suh L.A., Carter R., Norton J., Grammer L.C., Cho S.H., Tan B.K. (2013). Excessive fibrin deposition in nasal polyps caused by fibrinolytic impairment through reduction of tissue plasminogen activator expression. Am. J. Respir. Crit. Care Med..

[80] Takabayashi T., Kato A., Peters A.T., Hulse K.E., Suh L.A., Carter R., Norton J., Grammer L.C., Tan B.K., Chandra R.K. (2013). Increased expression of factor XIII-A in patients with chronic rhinosinusitis with nasal polyps. J. Allergy Clin. Immunol..

[81] Pollard J.W. (2004). Tumour-educated macrophages promote tumour progression and metastasis. Nat. Rev. Cancer.

[82] Bárdos H., Molnár P., Csécsei G., Adány R. (1996). Fibrin deposition in primary and metastatic human brain tumours. Blood Coagul. Fibrinolysis Int. J. Haemost. Thromb..

[83] Bárdos H., Juhász A., Répássy G., Adány R. (1998). Fibrin deposition in squamous cell carcinomas of the larynx and hypopharynx. Thromb. Haemost..

[84] Hao N.B., Lü M.H., Fan Y.H., Cao Y.L., Zhang Z.R., Yang S.M. (2012). Macrophages in tumor microenvironments and the progression of tumors. Clin. Dev. Immunol..

[85] Adány R. (1993). Janus-faced tumor-associated macrophages. Immunol. Today.

[86] Palumbo J.S., Barney K.A., Blevins E.A., Shaw M.A., Mishra A., Flick M.J., Kombrinck K.W., Talmage K.E., Souri M., Ichinose A. (2008). Factor XIII transglutaminase supports hematogenous tumor cell metastasis through a mechanism dependent on natural killer cell function. J. Thromb. Haemost..

[87] Vairaktaris E., Vassiliou S., Yapijakis C., Spyridonidou S., Vylliotis A., Derka S., Nkenke E., Fourtounis G., Neukam F.W., Patsouris E. (2007). Increased risk for oral cancer is associated with coagulation factor XIII but not with factor XII. Oncol. Rep..

[88] Song Y., Sakamoto F., Ito M. (2005). Characterization of factor XIIIa+ dendritic cells in dermatofibroma: Immunohistochemical, electron and immunoelectron microscopical observations. J. Dermatol. Sci..

[89] Cerio R., Spaull J., Jones E.W. (1989). Histiocytoma cutis: A tumour of dermal dendrocytes (dermal dendrocytoma). Br. J. Dermatol..

[90] Abenoza P., Lillemoe T. (1993). CD34 and factor XIIIa in the differential diagnosis of dermatofibroma and dermatofibrosarcoma protuberans. Am. J. Dermatopathol..

[91] Goldblum J.R., Tuthili R.J. (1997). CD34 and factor-XIIIa immunoreactivity in dermatofibrosarcoma protuberans and dermatofibroma. Am. J. Dermatopathol..

[92] Hsi E.D., Nickoloff B.J. (1996). Dermatofibroma and dermatofibrosarcoma protuberans: An immunohistochemical study reveals distinctive antigenic profiles. J. Dermatol. Sci..

[93] Invernizzi R., De Fazio P., Iannone A.M., Zambelli L.M., Rastaldi M.P., Ippoliti G., Ascari E. (1992). Immunocytochemical detection of factor XIII A--subunit in acute leukemia. Leuk. Res..

[94] Adany R., Kiss A., Muszbek L. (1987). Factor XIII: A marker of mono- and megakaryocytopoiesis. Br. J. Haematol..

[95] Kiss F., Hevessy Z., Veszprémi A., Katona E., Kiss C., Vereb G., Muszbek L., Kappelmayer J.N. (2006). Leukemic lymphoblasts, a novel expression site of coagulation factor XIII subunit A. Thromb. Haemost..

[96] Gyurina K., Kárai B., Ujfalusi A., Hevessy Z., Barna G., Jáksó P., Pálfi-Mészáros G., Póliska S., Scholtz B., Kappelmayer J. (2019). Coagulation fxiii-a protein expression defines three novel sub-populations in pediatric b-cell progenitor acute lymphoblastic leukemia characterized by distinct gene expression signatures. Front. Oncol..

[97] Guerrouahen B.S., Al-Hijji I., Tabrizi A.R. (2011). Osteoblastic and vascular endothelial niches, their control on normal hematopoietic stem cells, and their consequences on the development of leukemia. Stem Cells Int..

[98] Harris N.L., Dvorak A.M., Smith J., Dvorak H.F. (1982). Fibrin deposits in Hodgkin’s disease. Am. J. Pathol..

[99] Ádány R., Nemes Z., Muszbek L. (1987). Characterization of factor XIII containing-macrophages in lymph nodes with hodgkin’s disease. Br. J. Cancer.

[100] Naukkarinen J., Surakka I., Pietilainen K.H., Rissanen A., Salomaa V., Ripatti S., Yki-Jarvinen H., Duijn C.M., Wichmann H.E., Kaprio J. (2010). Use of genome-wide expression data to mine the “gray zone” of GWA studies leads to novel candidate obesity genes. PLoS Genet..

[101] Kaartinen M.T., Arora M., Heinonen S., Hang A., Barry A., Lundbom J., Hakkarainen A., Lundholm N., Rissanen A., Kaprio J. (2020). F13A1 transglutaminase expression in human adipose tissue increases in acquired excess weight and associates with inflammatory status of adipocytes. Int. J. Obes..

[102] Sottile J., Hocking D.C., Swiatek P.J. (1998). Fibronectin matrix assembly enhances adhesion-dependent cell growth. J. Cell Sci..

[103] Sottile J., Hocking D.C. (2002). Fibronectin polymerization regulates the composition and stability of extracellular matrix fibrils and cell-matrix adhesions. Mol. Biol. Cell.

[104] Wang Y., Zhao L., Smas C., Sul H.S. (2010). Pref-1 Interacts with Fibronectin To Inhibit Adipocyte Differentiation. Mol. Cell. Biol..

[105] Lee S.H., Park H.S., Lee J.A., Song Y.S., Jang Y.J., Kim J.H., Lee Y.J., Heo Y. (2013). Fibronectin gene expression in human adipose tissue and its associations with obesity-related genes and metabolic parameters. Obes. Surg..

[106] Mosher D.F., Schad P.E., Kleinman H.K. (1979). Cross-linking of fibronectin to collagen by blood coagulation factor XIIIa. J. Clin. Investig..

[107] Myneni V.D., Mousa A., Kaartinen M.T. (2016). Factor XIII-A transglutaminase deficient mice show signs of metabolically healthy obesity on high fat diet. Sci. Rep..

[108] Procyk R., Blomback B. (1988). Factor XIII-induced crosslinking in solutions of fibrinogen and fibronectin. BBA Gen. Subj..

[109] Sane D.C., Moser T.L., Pippen A.M.M., Parker C.J., Achyuthan K.E., Greenberg C.S. (1988). Vitronectin is a substrate for transglutaminases. Biochem. Biophys. Res. Commun..

[110] Prince C.W., Dickie D., Krumdieck C.L. (1991). Osteopontin, a substrate for transglutaminase and Factor XIII activity. Biochem. Biophys. Res. Commun..

[111] Lynch G.W., Slayter H.S., Miller B.E., McDonagh J. (1987). Characterization of thrombospondin as a substrate for factor XIII transglutaminase. J. Biol. Chem..

[112] Inbal A., Oldenburg J., Carcao M., Rosholm A., Tehranchi R., Nugent D. (2012). Recombinant factor XIII: A safe and novel treatment for congenital factor XIII deficiency. Blood.

[113] Schmitz T., Bäuml C.A., Imhof D. (2020). Inhibitors of blood coagulation factor XIII. Anal. Biochem..

